# Trends in Planetary Health Diet Index for the United States (PHDI‐US) Scores and Associations With Mortality Risk in the United States Between 1999 and 2020

**DOI:** 10.1002/fsn3.71985

**Published:** 2026-06-11

**Authors:** Tongle Yin, Mengshan Pan, ZiYing Jiang, Molly K. Parker, Jiamin Xu, Valisa E. Hedrick, Feiyun Zhu, Ying Yang, Rucheng Chen, Weijun Zheng

**Affiliations:** ^1^ School of Public Health, Zhejiang Chinese Medical University Hangzhou Zhejiang China; ^2^ Department of Human Nutrition, Foods, and Exercise Virginia Tech Blacksburg Virginia USA

**Keywords:** mortality risk, NHANES, PHDI‐US, trends analysis

## Abstract

This study utilized the Planetary Health Diet Index for the United States (PHDI‐US), a dietary index developed and validated for the U.S. population, to characterize temporal trends in PHDI‐US scores from 1999 to 2020, evaluate adherence to the EAT‐Lancet Planetary Health Diet, and examine associations between PHDI‐US scores and mortality risk. PHDI‐US scores were calculated using data from the National Health and Nutrition Examination Survey (NHANES) spanning 1999 to 2020, which encompassed a cohort of 55,579 adults ≥ 19 years of age living in the U.S. Total PHDI‐US scores can range between 0 and 150 and were derived from 24‐h dietary recalls. Weighted linear regression was used for trend analysis, and weighted Cox regression was employed to study mortality risk. Analysis of the aggregate dataset revealed a significant upward trend (*p*‐trend < 0.001) in PHDI‐US scores over the 21‐year period. Over this time, the average total PHDI‐US score for the overall U.S. population ranged between 41.2 and 46.5. This increasing trend was mirrored across various subpopulations, including age brackets, gender, race/ethnicity, poverty‐income ratios and educational levels. Higher scores (Q4 vs. Q1) were inversely associated with all‐cause mortality (HR = 0.78, 95% CI: 0.70–0.88, *p* < 0.001; consistent across all subgroups), cardiovascular mortality (HR = 0.77, 95% CI: 0.61–0.98; *p* = 0.034), and cerebrovascular mortality (*p*‐trend = 0.026). This study found a low total PHDI‐US score and a progressive escalation in scores among the American population between 1999 and 2020, a pattern also evident across all evaluated subgroups. In addition, an inverse association was discerned between PHDI‐US scores and mortality risk, indicating that elevated PHDI‐US scores might be concomitant with reduced mortality risk. This study has important public health implications for optimizing dietary patterns in the United States.

## Graphical abstract

Graphical abstract of the study design, PHDI‐US adherence characteristics, and associations with mortality risk among U.S. adults in NHANES 1999–2020.

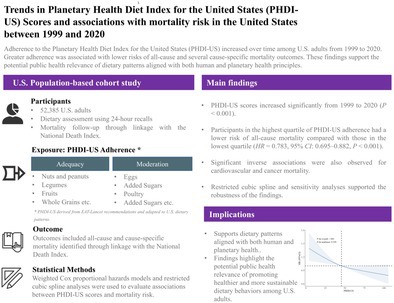

**Abbreviations**
AHEI: Alternative Healthy Eating Index
AMED: Alternate Mediterranean Diet Score
ANOVA: analysis of variance
BMI: body mass index
COVID‐19: coronavirus disease 2019
DALYs: disability‐adjusted life years
DII: Dietary Inflammation Index
FPEs: food portion equivalents
HEI‐2015: Healthy Eating Index‐2015
HPDI: Healthful Plant‐Based Diet Index
HR: hazard ratio
NCHS: National Center for Health Statistics
NDI: National Death Index
NHANES: National Health and Nutrition Examination Survey
PHD: planetary health diet
PHDI: Planetary Health Diet Index
PHDI‐US: Planetary Health Diet Index for the United States
PIR: poverty‐income ratio
SE: standard error
USDA: United States Department of Agriculture

## 1 Introduction

Diet is a critical determinant of health (GBD 2017 Diet Collaborators 2019), contributing to 22% of global adult deaths and 15% of adult disability‐adjusted life years (DALYs) through dietary risks, as quantified by the Global Burden of Disease Study (Downer et al. 2020). In the United States, diet‐related chronic diseases afflict over 60% of adults (Snetselaar et al. 2021), indicating that dietary patterns can have a major impact on mortality and disability‐adjusted life years (US Burden of Disease Collaborators et al. 2018). Consequently, a better understanding of the relationship between diet quality and mortality is imperative for devising strategies to improve public health.

Extensive research has been conducted to examine dietary pattern trends and their association with mortality. For instance, Shan et al. (2023) identified a notable inverse relationship between adherence to varied dietary patterns (including the Dietary Guidelines for Americans and the Mediterranean diet) and overall mortality. Individuals in the highest quintile of dietary scores (HEI‐2015, AMED, HPDI, and AHEI) had 14%–20% lower risk of all‐cause mortality compared to the lowest quintile, with consistent trends across demographic subgroups. In addition, Sotos‐Prieto et al. (2017) reported that participants with the greatest improvement in diet quality over 12 years experienced 9%–16% lower all‐cause mortality risk compared to those with stable diets.

However, despite evidence of gradual improvements over time, the overall diet quality in the U.S. population still falls substantially short of recommended levels, with persistent disparities across sociodemographic groups, with Popkin et al. (1996) having employed the Diet Quality Index to monitor dietary changes in a representative U.S. cohort from 1965 to 1991 (7.4–6.4), noting significant improvements in dietary quality over this time, yet on average, still not meeting the healthful diet score of 4 or less. Additional evidence also points to an ascending trajectory in U.S. adults' diet quality (Rehm et al. 2016; Shan et al. 2019); however, overall diets among the U.S. population are still far from meeting recommendations, and there are disparities based on age, socioeconomic status, ethnicity, and survey cycles (2001–2002 to 2017–2018) (Long et al. 2022; Orr et al. 2019).

In addition to impacting human health, food choices can also affect the environment and contribute to climatic challenges (Poore and Nemecek 2018). Dietary patterns based on animal products, especially meat from ruminant animals, require greater natural resources and produce more greenhouse gases in comparison to plant‐based dietary patterns (Clune et al. 2017; Scarborough et al. 2023).

This interconnection between diet, health, and sustainability has prompted the development of integrated dietary frameworks. In response to the escalating global demographic trend (Population Reference Bureau 2024) and the necessity to generate adequate food supplies (Willett et al. 2019), the adoption of more sustainable dietary practices is imperative. The EAT‐Lancet Commission's “Planetary Healthy Diet” is a proposed dietary pattern that aligns with both health and sustainability goals. This dietary pattern retains the principles proposed by the EAT‐Lancet Commission, which consider not only human health but also the environmental and climatic impacts of dietary patterns within the broader context of planetary health. It emphasizes high intake of vegetables, fruits, and whole grains and lower consumption of meats, fish, eggs, refined grains, and tubers (Willett et al. 2019). A longitudinal Swedish study associated adherence to this diet with substantial reductions in all‐cause (HR, 0.75), cancer (HR, 0.76), and cardiovascular (HR, 0.68) mortalities (Stubbendorff et al. 2022). The EPIC cohort study posited that full adherence to the Planetary Health Diet could significantly curtail death (19%–63%) and cancer (10%–39%) incidence rates over two decades (Laine et al. 2021).

To measure adherence to the Planetary Health Diet, Cacau et al. (2021) developed the Planetary Health Diet Index (PHDI) using data from the Brazilian Longitudinal Study of Adult Health (ELSA‐brasil). The PHDI has since been both adapted and applied in various global contexts. Ye et al. (2023) found that higher adherence to the Planetary Health Diet was associated with reduced risk of chronic disease mortality using Singaporean data. Furthermore, Cacau et al. (2024) demonstrated that greater adherence to the Planetary Health Diet, as measured by PHDI scores, among European adolescents may be associated with improved cardiovascular health outcomes.

Previous studies on the PHDI have shown that greater adherence to the PHDI is associated with a reduced risk of mortality. However, these findings may not be fully generalizable to the U.S. population. To examine temporal trends in PHDI scores and their association with mortality among U.S. adults, Parker et al. (2023) adapted the original Planetary Health Diet Index scoring system (Cacau et al. 2021) and used data from the National Health and Nutrition Examination Survey (NHANES) to develop and validate the U.S. version of the Planetary Health Diet Index (PHDI‐US). This adaptation provides a tool to assess U.S. adherence to the Planetary Health Diet and enables exploration of dietary trends across diverse demographics (Cacau et al. 2021), which is important given the evidence of disparities in dietary quality by income, age, race/ethnicity, and gender (Willett et al. 2019). Some advantages of utilizing the PHDI‐US over other proposed indices include a scoring system that uses continuous values, is relative to total individual consumption (percentage of total grams of foods and beverages consumed), and does not inherently deduct points for vegan and vegetarian dietary patterns that exclude fish and/or eggs (Willett et al. 2019).

PHDI‐US provides a unified metric that explicitly addresses the dual goals of human health and environmental sustainability. Unlike established indices such as the HEI, which are primarily designed to assess nutritional adequacy and chronic disease prevention, the PHDI‐US is fundamentally structured around the framework of the EAT‐Lancet Commission. Its core advantage lies in its systematic quantification of how well dietary patterns align with both personal health benefits and planetary boundaries, particularly in evaluating the environmental impact of food choices, such as red meat consumption. While traditional indices may yield incidental environmental benefits, the PHDI‐US makes sustainable food systems a central and measured objective within its scoring criteria.

Although the utility and validity of the PHDI‐US have been supported (Parker et al. 2023), comprehensive evidence regarding the long‐term trends in PHDI‐US scores within the U.S. population and their association with all‐cause mortality remains lacking. Investigating this relationship will provide valuable insight into adherence to the Planetary Health Diet and its potential link to mortality risk. Understanding these patterns is vital for determining the health benefits associated with the dietary recommendations from EAT‐Lancet. This study used data from the NHANES cycles spanning 1999–2000 through 2017–2020 to characterize temporal trends in PHDI‐US scores among U.S. population, examine the association between PHDI‐US scores and mortality risk, and assess the broader public health implications of dietary patterns for mortality reduction and the promotion of sustainable food systems.

## 2 Methods

### 2.1 Data Source and Population

**FIGURE 1:**
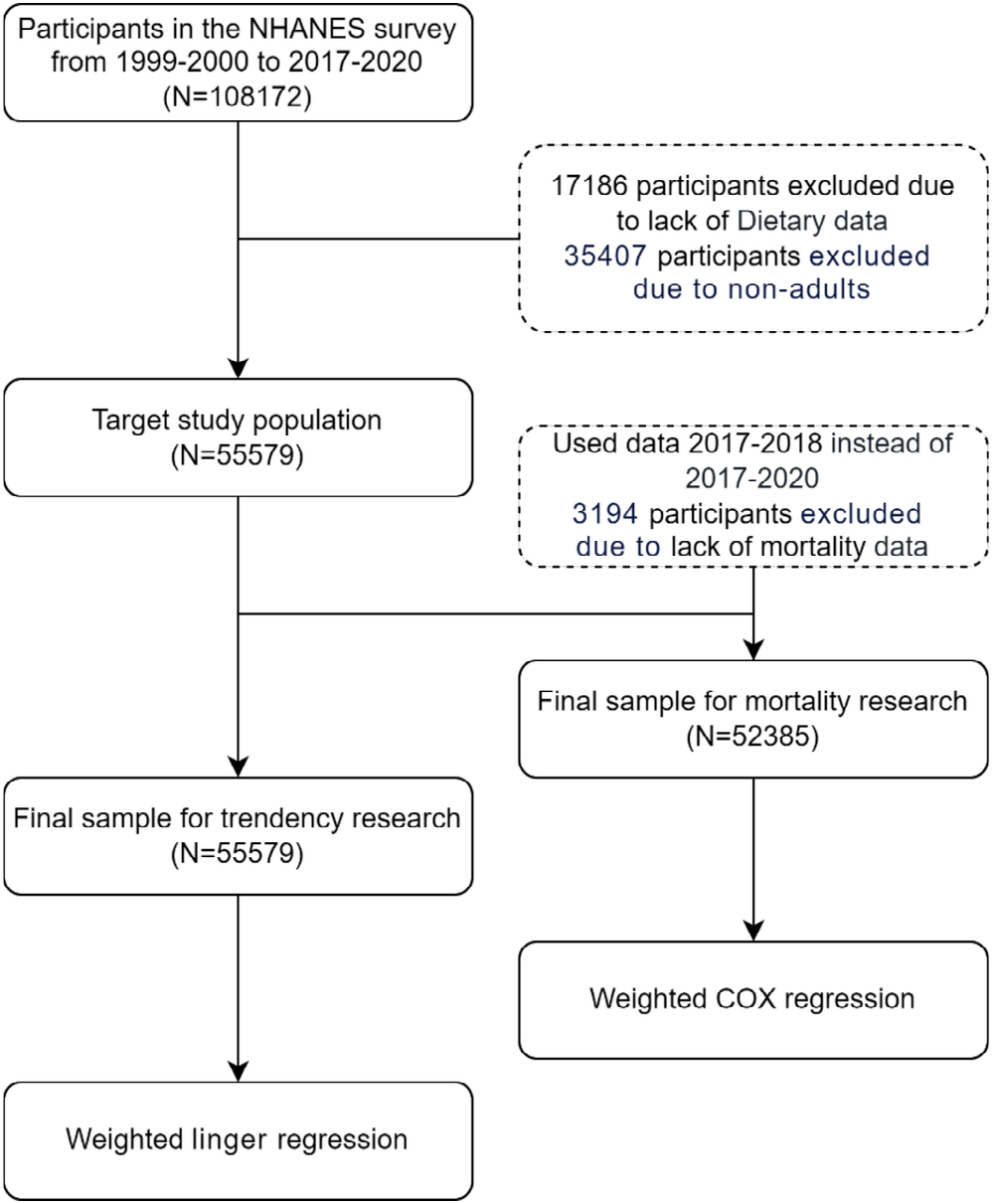
Participant inclusion and exclusion flowchart.

This study utilized data from eight National Health and Nutrition Examination Survey (NHANES) cycles spanning 1999–2020. To ensure national representativeness, data from the incomplete 2019–2020 cycle were combined with the 2017–2018 cycle to form a pre‐pandemic sample (2017‐March 2020). Consequently, analyses of baseline characteristics and secular trends were conducted using data from 1999 to 2020, while mortality analyses were restricted to data from 1999 to 2018 with follow‐up through 2019. The study flow is presented in Figure 1. The baseline characteristics of the participants are summarized in Table 1, which includes key variables across the following domains: sociodemographic characteristics: age, gender, race/ethnicity, education level, poverty‐income ratio (PIR), and marital status; lifestyle factors: total calorie intake, smoking status, alcohol consumption, and physical activity level; and health status: body mass index (BMI) and the presence of underlying diseases, including hypertension, diabetes, and cardiovascular diseases. Detailed definitions and classifications for all variables are provided in Table 1. All participants provided informed consent in accordance with relevant guidelines, and the data are publicly available on the NHANES website (https://www.cdc.gov/nchs/nhanes/index.htm).

**TABLE 1:** Baseline characteristics of participants stratified by PHDI‐US score quartiles.

Variable	Total (*n* = 55,579)	Score group				*p*
		Q1 (*n* = 13,895)	Q2 (*n* = 13,895)	Q3 (*n* = 13,894)	Q4 (*n* = 13,895)	
Age, mean ± SE	46.06 ± 0.2	42.76 ± 0.23	44.58 ± 0.25	46.74 ± 0.3	49.66 ± 0.31	< 0.001
Total calorie intake (kcal/d), mean (SE)	2120.23 ± 6.5	2194.51 ± 11.02	2195.22 ± 12.67	2117.5 ± 11.95	1987.32 ± 11.28	< 0.001
PIR, mean ± SE	28.71 ± 0.06	29.08 ± 0.09	29.1 ± 0.09	28.76 ± 0.11	27.98 ± 0.1	< 0.001
BMI, mean ± SE	2.97 ± 0.03	2.57 ± 0.03	2.84 ± 0.03	3.08 ± 0.03	3.33 ± 0.03	< 0.001
Gender, *n* (survey‐weighted %)						
Male	26,903 (48.28)	7784 (55.67)	7072 (51.9)	6390 (46.22)	5657 (40.45)	< 0.001
Female	28,676 (51.72)	6111 (44.33)	6823 (48.1)	7504 (53.78)	8238 (59.55)	
Race, *n* (survey‐weighted %)						
Mexican American	9932 (8.33)	2617 (9.23)	2574 (8.79)	2578 (8.6)	2163 (6.85)	< 0.001
Hispanic	4576 (5.68)	1048 (6.34)	1128 (5.9)	1164 (5.47)	1236 (5.1)	
Non‐Hispanic white	23,992 (67.56)	5280 (61.37)	6127 (67.81)	6369 (70.1)	6216 (70.18)	
Non‐Hispanic black	12,237 (11.42)	4182 (17.38)	3159 (11.9)	2699 (9.69)	2197 (7.5)	
Other	4842 (7.02)	768 (5.69)	907 (5.6)	1084 (6.13)	2083 (10.37)	
EDU, *n* (survey‐weighted %)						
Less than ninth grade	5925 (5.52)	1617 (6.9)	1446 (5.58)	1496 (5.49)	1366 (4.33)	< 0.001
9–11th grade (Includes 12th grade with no diploma)	7588 (11.1)	2540 (16.87)	2014 (12.11)	1694 (9.12)	1340 (7.23)	
High school graduate/general equivalency diploma	12,048 (24.33)	3412 (29.68)	3245 (27.46)	2917 (23.65)	2474 (17.6)	
Some college or associates degree	14,822 (31.12)	3536 (30.98)	3802 (32.89)	3852 (31.91)	3632 (28.84)	
College graduate or higher	11,258 (27.93)	1454 (15.57)	2246 (21.96)	2977 (29.82)	4581 (41.99)	
Marital, *n* (survey‐weighted %)						
Living with partner	30,926 (61.64)	7055 (56.4)	7479 (60.04)	7889 (62.99)	8503 (66.27)	< 0.001
Not living with partner	22,367 (38.36)	6146 (43.6)	5742 (39.96)	5450 (37.01)	5029 (33.73)	
Smoke, *n* (survey‐weighted %)						
No	28,955 (54.13)	6101 (47.1)	6849 (51.57)	7496 (55.68)	8509 (60.94)	< 0.001
Past	12,879 (24.66)	2820 (21.04)	3141 (23.49)	3297 (24.91)	3621 (28.57)	
Now	10,769 (21.21)	3908 (31.86)	3061 (24.93)	2389 (19.41)	1411 (10.5)	
Alcohol drink, *n* (survey‐weighted %)						
No	13,595 (22.18)	3089 (21.68)	3221 (21.36)	3440 (21.87)	3845 (23.67)	0.018
Yes	36,517 (77.82)	9139 (78.32)	9245 (78.64)	9162 (78.13)	8971 (76.33)	
PA, *n* (survey‐weighted %)						
Inactive	18,208 (34.67)	4461 (37)	4556 (36.06)	4685 (35.56)	4506 (30.83)	< 0.001
Active	28,967 (65.33)	6616 (63)	7060 (63.94)	7257 (64.44)	8034 (69.17)	
Underlying diseases, *n* (survey‐weighted %)						
No	34,082 (72.44)	8279 (72.95)	8591 (73)	8505 (72.38)	8707 (71.56)	0.016
Yes	17,578 (27.56)	4309 (27.05)	4157 (27)	4435 (27.62)	4677 (28.44)	

*Note:* Quartiles divide a dataset into four equal parts. The first quartile (Q1) is the median of the lower half of the data, and the third quartile (Q3) is the median of the upper half of the data. The second quartile is the same as the median. Underlying diseases: Includes diabetes, hypertension, and cardiovascular diseases.

Abbreviations: BMI, body mass index; EDU, education level; PA, physical activity; PHDI‐US, Planetary Health Diet Index for the United States; PIR, poverty income ratio.

### 2.2 Determination of Dietary Intake

NHANES collects dietary data through computer‐assisted personal interviews in mobile examination centers (He et al. 2021). The study utilized the average of two nonconsecutive 24‐h dietary recalls (Day 1 and Day 2) when available, while also retaining participants with only single‐day recall data to maximize sample representativeness of the U.S. population. Single‐day recall: (1) Participants from the 1999 to 2002 survey cycles (only Day 1 data). (2) Subsequent survey participants who did not complete the Day 2 follow‐up. The 24‐h recall is a validated approach for estimating population dietary trends (Zipf et al. 2013).

### 2.3 Calculation of PHDI‐US Score

The PHDI‐US score quantifies alignment with the Planetary Health Diet, with total scores ranging from 0 to 150 (higher scores indicate greater adherence). It is derived from 16 dietary components, categorized into four groups based on intake goals: (1) Adequacy (score increases as intake approaches recommended amounts, e.g., fruits); (2) Optimum (score increases toward, then decreases beyond an optimal level, e.g., nuts); (3) Ratio (score reflects diversity within food groups, e.g., proportion of non‐starchy vegetables); and (4) Moderation (score decreases after exceeding recommended limits, e.g., red meat). Scores for most components are calculated as a percentage of total dietary weight (grams and excluding water), with gram quantities derived from food portion equivalents using the conversion factors established by Blackstone and Conrad (2020). A detailed description follows.

### 2.4 Detailed Methodology

**FIGURE 2:**
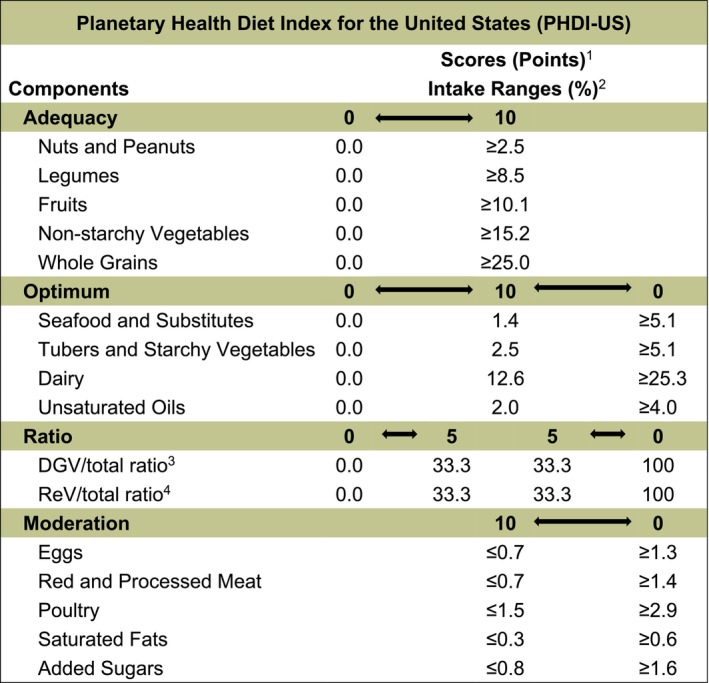
The planetary health diet index for the United States (PHDI‐US). Components and scoring criteria. ^1^Scores range between 0 and 10 for adequacy, optimum, and moderation components. Scores range between 0 and 5 for ratio components. Arrows indicate where scores increase or decrease proportionally for the corresponding intake ranges. ^2^Component intake ranges are represented as the percentage of total grams of foods and beverages (excluding water) consumed. ^3^DGV/total ratio component is the ratio between the grams of dark green vegetables (DGV) consumed and the grams consumed within the non‐starchy vegetables component. Intake range is represented as a percentage of non‐starchy vegetables consumed. ^4^ReV/total ratio component is the ratio between the grams of red and orange vegetables (ReV) consumed and the grams consumed within the non‐starchy vegetables component. Intake range is represented as a percentage of non‐starchy vegetables consumed.

The PHDI‐US scoring system includes 16 components, with scores ranging between 0 and 150, with higher scores indicating greater dietary adherence to recommendations (Parker et al. 2023) (Figure 2). Components are classified into four categories: adequacy, optimum, ratio, and moderation, based on the original PHDI (Cacau et al. 2021) and Planetary Health Diet recommendations (Willett et al. 2019). Scores are computed by evaluating the percentage intake for each component relative to the total grams of foods and beverages (excluding water) consumed, with the exception of ratio components being relative to total grams of non‐starchy vegetable intake to measure diversity of vegetable consumption. For the adequacy components, scores are increased proportionally when intake approaches the adequate intake recommendation. For optimum components, scores are increased proportionally when intake approaches the optimal intake recommendation and then decrease proportionally when exceeding that level. For moderation components, scores decrease proportionally after exceeding the moderate intake recommendation. We employed the conversion factors established by Blackstone and Conrad (2020) to transform the original food portion equivalents (FPEs) in NHANES data into gram quantities.

Categorization of foods into each PHDI‐US component is based on the USDA food pattern equivalents (FPE) that are defined in the Food Pattern Equivalents database (WWEIA/NHANES Overview 2025) for each NHANES cycle (Parker et al. 2023). The FPE were mapped to each component and used to classify foods reported during 24‐h recalls. For example, the Non‐starchy vegetables component consists of foods from three FPE: dark green vegetables, total red and orange vegetables, and other vegetables. The comprehensive methodology for the PHDI‐US score calculation and food categorization is detailed by Parker et al. (2023).

### 2.5 Determination of Mortality

The focus of the mortality analysis was all‐cause mortality risk (alive as MORTSTAT = 0) and attributable mortality risk, accessible via (https://www.cdc.gov/nchs/data‐linkage/mortality.htm). Underlying cause‐specific mortality was defined as deaths attributed to one of the 10 leading causes, classified by the NCHS 2019 UCOD_LEADING variable.

### 2.6 Statistical Methods

Analytical procedures accounted for the NHANES complex sampling framework to ensure nationally representative estimates. Both overall and component PHDI‐US scores, in addition to population averages for major food and nutrient intakes, were computed for each NHANES cycle.

Baseline data were stratified into quartiles based on PHDI‐US scores. Quantitative data conformed to normality assumptions and were presented as mean ± standard error (SE), while qualitative data were expressed in frequency (percentage). To assess significant differences in factors across the four groups of the PHDI‐US score, weighted ANOVA was conducted for continuous variables, and weighted chi‐square tests were applied for categorical variables.

Trends were analyzed using survey‐weighted linear regression, treating survey years as continuous variables. Trend analysis has been adjusted for age and gender. Absolute and relative differences between the estimated means (95% CI) from 1999 to 2000 to 2017 to 2020 were calculated, considering each biennial cycle as a distinct category. Subgroup analyses were performed with adjustments for demographic variables, including gender, age, race/ethnicity, education level, and PIR. In the sensitivity analysis, we further adjusted for total calorie intake.

Mortality risks were evaluated using survey‐weighted Cox proportional hazards regression models. PHDI‐US scores were analyzed both as a continuous variable and as categorical variables based on quartiles. The association was examined through a series of multivariable models with sequential adjustments: The univariable model was not adjusted for any covariates; Model 1 was adjusted for age, gender, race/ethnicity, education level, PIR, and marital status; Model 2 was further adjusted for total calorie intake, smoking status, alcohol consumption, and physical activity level; Model 3 was additionally adjusted for BMI and the presence of underlying diseases (including hypertension, diabetes, and cardiovascular diseases). Fully adjusted subgroup analyses were conducted with the aforementioned demographic variables. Interaction effects between scores and demographic groups were probed using product interaction terms. Sensitivity analyses included (1) MICE multiple data imputation, with the imputation method selected by default based on data type. (2) To account for the latency period between dietary exposure and mortality outcomes, participants with less than 12 months of follow‐up were excluded, as the influence of diet on mortality is typically a long‐term process and deaths occurring within a short period may introduce incidental bias. (3) To minimize the potential impact of extreme values, we winsorized the PHDI‐US scores at the 2nd and 98th percentiles. Specifically, values below the 2nd percentile were set to the 2nd percentile value, and values above the 98th percentile were set to the 98th percentile value.

Statistical significance was established at *p* < 0.05. All statistical analyses were executed using R software, version 4.4.0, along with Zstats v1.0 (www.zstats.net).

## 3 Results

### 3.1 Participant Characteristics

From the 108,172 participants of the NHANES surveys (1999–2020), 55,579 participants were included, with 17,186 excluded due to incomplete or unreliable dietary data and 35,407 excluded due to Age < 18 (Figure 1). Stratification into four quartiles based on PHDI‐US scores (Q1: 0.07–36.7, Q2: 36.7–45.6, Q3: 45.6–55.4, and Q4: 55.4–117.9) is detailed in Table 1. Baseline demographics, including age, gender, race, education level, lifestyle choices (smoking and alcohol consumption), and health‐related metrics (BMI, PIR), varied significantly across these groups. The mean age was 46.06 years (SE, 0.20), with females constituting 51.72% of the sample. Racial composition included 67.56% non‐Hispanic White, 11.42% non‐Hispanic Black, 5.68% Hispanic, and 8.33% Mexican American. Educational attainment and lifestyle habits were also reported, with 31.12% holding university degrees and 27.93% having higher education, 54.13% nonsmokers, and 22.18% non‐alcohol consumers.

### 3.2 Trends in PHDI‐US Scores and Population Subgroups

**FIGURE 3:**
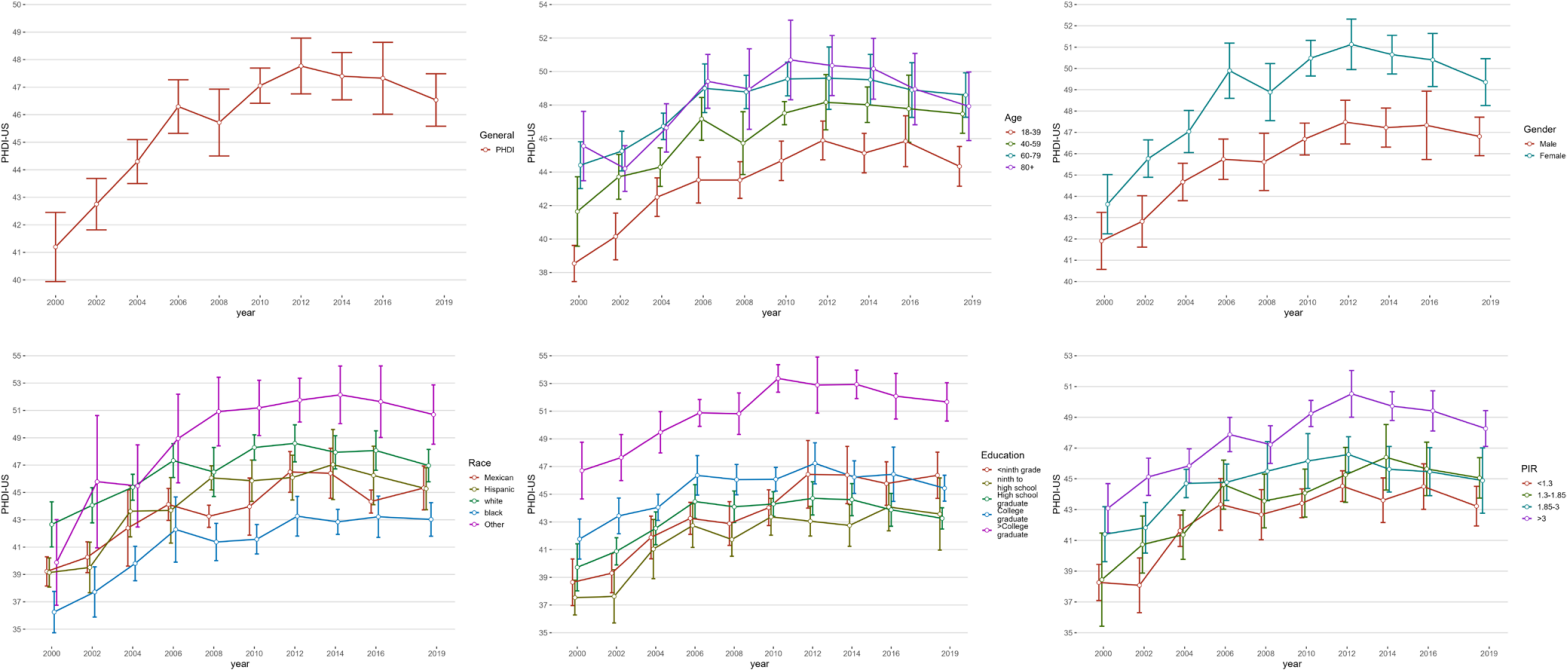
Trend graph of PHDI‐US scores over the years for the general population and subgroup populations.

A significant uptrend in PHDI‐US scores was observed from 1999 to 2020, rising from 41.2 to 46.5, *p* < 0.001, representing a 13.8% increment out of 150 points. The largest significant increases in scores over the 21‐year period were among those with other race/ethnicity individuals (30.1%), non‐Hispanic black individuals (19.6%), less than ninth grade individuals (18.9%), and those with a PIR between 1.3 and 1.85 (2.6%). Age‐related changes were significant in groups 40–59. These trends are quantified in Table 2 and visually presented in Figure 3.

**TABLE 2:** Trends in PHDI‐US scores across years and strata.

	1999–2000	2001–2002	2003–2004	2005–2006	2007–2008	2009–2010	2011–2012	2013–2014	2015–2016	2017–2020	Differences 2017–2020 vs. 1999–2000 (95% CI)	*p* for trend
General population	41.2 (39.9, 42.5)	42.8 (41.8, 43.7)	44.3 (43.5, 45.1)	46.3 (45.3, 47.3)	45.7 (44.5, 46.9)	47.1 (46.4, 47.7)	47.8 (46.8, 48.8)	47.4 (46.5, 48.3)	47.3 (46.0, 48.6)	46.5 (45.6, 47.5)	5.7 (4.1, 7.3)	< 0.001
Gender												
Male	41.9 (40.6, 43.2)	42.8 (41.6, 44.0)	44.7 (43.8, 45.5)	45.7 (44.8, 46.7)	45.6 (44.3, 47.0)	46.7 (45.9, 47.4)	47.5 (46.5, 48.5)	47.2 (46.3, 48.1)	47.3 (45.7, 48.9)	46.8 (45.9, 47.7)	5.3 (3.6, 7.0)	< 0.001
Female	43.6 (42.2, 45.0)	45.8 (44.9, 46.6)	47.0 (46.1, 48.0)	49.9 (48.6, 51.2)	48.9 (47.5, 50.2)	50.5 (49.6, 51.3)	51.1 (49.9, 52.3)	50.6 (49.7, 51.6)	50.4 (49.2, 51.6)	49.4 (48.3, 50.5)	6.1 (4.3, 8.0)	< 0.001
Race/ethnicity												
Mexican American	39.2 (38.2, 40.3)	40.3 (39.1, 41.4)	42.4 (39.6, 45.2)	44.1 (43.0, 45.3)	43.3 (42.4, 44.1)	44.0 (41.9, 46.1)	46.5 (45.0, 48.0)	46.4 (44.6, 48.2)	44.3 (43.5, 45.2)	45.3 (43.7, 46.9)	6.2 (4.2, 8.1)	< 0.001
Hispanic	39.1 (38.1, 40.2)	39.5 (37.7, 41.4)	43.6 (41.8, 45.5)	43.7 (41.3, 46.1)	46.1 (45.2, 46.9)	45.9 (44.3, 47.4)	46.1 (44.4, 47.7)	47.0 (44.5, 49.6)	46.2 (44.1, 48.4)	45.3 (43.7, 46.8)	5.9 (3.8, 8.0)	< 0.001
Non‐Hispanic white	42.7 (41.0, 44.3)	44.1 (42.8, 45.3)	45.4 (44.4, 46.3)	47.3 (46.1, 48.6)	46.5 (44.7, 48.3)	48.3 (47.4, 49.2)	48.6 (47.2, 49.9)	47.9 (46.7, 49.1)	48.1 (46.6, 49.5)	47.0 (45.8, 48.2)	4.8 (2.7, 6.9)	< 0.001
Non‐Hispanic black	36.3 (34.7, 37.8)	37.7 (35.9, 39.6)	39.8 (38.5, 41.1)	42.3 (39.9, 44.7)	41.4 (40.0, 42.7)	41.6 (40.5, 42.7)	43.3 (41.8, 44.7)	42.8 (41.9, 43.8)	43.2 (41.7, 44.7)	43.0 (41.8, 44.3)	7.1 (5.1, 9.0)	< 0.001
Other	39.9 (36.8, 43.0)	45.8 (40.9, 50.6)	45.5 (42.5, 48.5)	48.9 (45.7, 52.2)	50.9 (48.4, 53.4)	51.2 (49.2, 53.2)	51.8 (50.1, 53.4)	52.1 (50.0, 54.3)	51.6 (49.0, 54.3)	50.7 (48.5, 52.9)	12.0 (8.3, 15.7)	< 0.001
Age group, years												
20–39	38.5 (37.5, 39.6)	40.1 (38.8, 41.5)	42.5 (41.3, 43.6)	43.5 (42.1, 44.9)	43.5 (42.4, 44.6)	44.7 (43.5, 45.8)	45.9 (44.7, 47.0)	45.1 (44.0, 46.3)	45.8 (44.3, 47.4)	44.3 (43.2, 45.5)	5.8 (4.1, 7.5)	< 0.001
40–59	41.6 (39.6, 43.7)	43.7 (42.4, 45.0)	44.3 (43.1, 45.4)	47.2 (45.9, 48.4)	45.7 (43.8, 47.6)	47.5 (46.8, 48.2)	48.2 (46.5, 49.8)	48.0 (47.0, 49.1)	47.8 (45.8, 49.8)	47.5 (46.3, 48.6)	5.8 (3.5, 8.1)	< 0.001
60–79	44.4 (43.0, 45.8)	45.3 (44.1, 46.4)	46.7 (45.9, 47.5)	49.0 (47.5, 50.5)	48.8 (47.8, 49.8)	49.6 (48.5, 50.6)	49.6 (47.7, 51.5)	49.5 (48.0, 51.0)	48.9 (47.3, 50.5)	48.6 (47.3, 49.9)	4.2 (2.2, 6.1)	< 0.001
> 80	45.5 (43.5, 47.6)	44.2 (42.8, 45.6)	46.6 (45.2, 48.1)	49.4 (47.8, 51.0)	49.0 (46.6, 51.4)	50.7 (48.3, 53.1)	50.4 (48.6, 52.2)	50.2 (48.4, 52.0)	48.9 (46.8, 51.1)	47.9 (45.9, 50.0)	2.2 (−0.6, 5.0)	0.002
Education												
Less than ninth grade	38.6 (37.0, 40.3)	39.3 (37.9, 40.7)	41.9 (40.3, 43.4)	43.3 (42.1, 44.4)	42.9 (41.3, 44.5)	44.0 (42.7, 45.3)	46.4 (44.0, 48.9)	46.4 (44.3, 48.4)	45.8 (44.2, 47.3)	46.4 (44.7,48.0)	7.3 (4.7, 9.9)	< 0.001
From ninth grade to high school graduation	37.5 (36.3, 38.8)	37.6 (35.7, 39.5)	41.0 (38.9, 43.2)	42.7 (41.2, 44.3)	41.7 (40.5, 42.9)	43.4 (42.0, 44.7)	43.1 (42.0, 44.1)	42.7 (41.2, 44.3)	44.1 (42.4, 45.8)	43.6 (41.0, 46.2)	6.5 (3.6, 9.4)	< 0.001
High school graduate	39.7 (38.0, 41.4)	40.9 (39.9, 41.9)	42.5 (41.4, 43.7)	44.5 (43.3, 45.7)	44.1 (43.0, 45.2)	44.3 (43.4, 45.2)	44.7 (43.5, 45.9)	44.6 (43.4, 45.8)	43.9 (42.7, 45.1)	43.3 (42.5, 44.0)	3.8 (1.7, 5.9)	< 0.001
College graduate	41.8 (40.3, 43.2)	43.4 (42.1, 44.7)	44.1 (43.1, 45.0)	46.4 (44.9, 47.8)	46.1 (45.0, 47.1)	46.1 (45.2, 46.9)	47.2 (45.7, 48.7)	46.2 (45.1, 47.4)	46.4 (44.5, 48.4)	45.4 (44.5, 46.4)	4.2 (2.4, 5.9)	< 0.001
Above College degree	46.7 (44.7, 48.8)	47.6 (46.0, 49.3)	49.5 (48.0, 51.0)	50.9 (49.9, 51.8)	50.8 (49.3, 52.3)	53.4 (52.4, 54.4)	52.9 (50.9, 54.9)	52.9 (51.9, 54.0)	52.1 (50.4, 53.7)	51.7 (50.3, 53.0)	5.6 (3.0, 8.1)	< 0.001
PIR												
< 1.3	38.3 (37.1, 39.4)	38.1 (36.3, 39.9)	41.6 (40.6, 42.6)	43.3 (41.7, 45.0)	42.7 (41.0, 44.3)	43.4 (42.5, 44.3)	44.5 (43.5, 45.5)	43.6 (42.2, 45.1)	44.5 (43.0, 46.0)	43.2 (41.9, 44.5)	4.9 (3.3, 6.6)	< 0.001
1.3–1.85	38.4 (35.4, 41.5)	40.7 (38.9, 42.6)	41.4 (39.8, 43.0)	44.6 (43.0, 46.2)	43.6 (41.8, 45.3)	44.1 (42.5, 45.6)	45.3 (43.5, 47.0)	46.4 (44.3, 48.5)	45.6 (43.9, 47.4)	45.1 (43.7, 46.4)	7.1 (3.8, 10.4)	< 0.001
1.85–3	41.4 (39.6, 43.2)	41.8 (40.2, 43.5)	44.7 (43.8, 45.6)	44.8 (43.6, 46.0)	45.5 (43.6, 47.4)	46.2 (44.4, 47.9)	46.6 (45.4, 47.7)	45.6 (44.1, 47.1)	45.5 (43.9, 47.0)	44.9 (42.8, 47.0)	4.0 (1.2, 6.8)	0.002
> 3	43.1 (41.5, 44.7)	45.1 (43.9, 46.3)	45.8 (44.7, 46.9)	47.9 (46.8, 49.0)	47.2 (46.0, 48.4)	49.2 (48.4, 50.1)	50.5 (49.0, 52.0)	49.7 (48.8, 50.7)	49.4 (48.1, 50.7)	48.3 (47.1, 49.4)	5.7 (3.7, 7.8)	< 0.001

**FIGURE 4:**
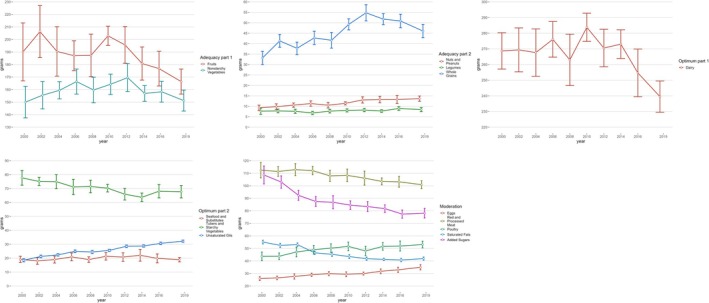
Trend graph of component grams of the PHDI‐US over the years.

The gram amounts of individual PHDI‐US components also showed notable variations over the study period, as detailed in Table 3 and illustrated in Figure 4. In sensitivity analyses, the trend of increasing PHDI‐US scores with year remained significant in the general population after adjustment for total calorie intake (*p* < 0.001) (Table S1).

**TABLE 3:** The component grams of the PHDI‐US over the years.

Components grams (SE)	1999–2000	2001–2002	2003–2004	2005–2006	2007–2008	2009–2010	2011–2012	2013–2014	2015–2016	2017–2020
Adequacy										
Nuts and Peanuts	9.3 (8.0, 10.5)	9.8 (8.5, 11.2)	10.6 (9.5, 11.6)	11.3 (10.0, 12.6)	10.5 (9.2, 11.9)	11.4 (10.7, 12.2)	13.0 (11.5, 14.5)	13.2 (11.7, 14.8)	13.2 (11.3, 15.2)	13.7 (12.4, 14.9)
Legumes	7.7 (6.2, 9.2)	7.8 (6.9, 8.7)	7.6 (6.6, 8.6)	6.8 (6.1, 7.5)	7.7 (6.8, 8.5)	8.0 (7.3, 8.8)	8.2 (7.4, 9.0)	7.7 (7.1, 8.4)	8.9 (8.1, 9.8)	8.4 (7.5, 9.4)
Fruits	190.1 (166.9, 213.2)	206.3 (185.6, 227.1)	190.5 (170.7, 210.2)	187.1 (175.1, 199.0)	187.3 (170.4, 204.3)	202.9 (195.2, 210.6)	195.6 (181.0, 210.3)	180.8 (167.6, 194.0)	176.7 (162.9, 190.6)	166.4 (156.4, 176.4)
Non‐starchy vegetables	150.0 (137.4, 162.6)	155.5 (144.6, 166.4)	159.4 (152.6, 166.3)	166.4 (156.4, 176.4)	159.7 (149.5, 169.9)	164.2 (156.1, 172.4)	169.6 (158.4, 180.7)	156.9 (150.6, 163.2)	158.3 (150.0, 166.6)	151.2 (142.8, 159.6)
Whole grains	33.2 (30.0, 36.3)	41.3 (38.3, 44.4)	37.8 (34.8, 40.7)	42.8 (39.6, 45.9)	41.6 (37.9, 45.3)	49.2 (46.6, 51.9)	54.8 (50.9, 58.7)	51.8 (49.1, 54.4)	50.9 (47.6, 54.1)	46.0 (42.8, 49.2)
Optimum										
Seafood and substitutes	19.1 (16.9, 21.4)	18.0 (15.6, 20.4)	19.0 (16.5, 21.5)	20.9 (18.1, 23.7)	19.0 (17.0, 21.0)	21.3 (18.6, 24.0)	20.8 (17.7, 23.8)	22.1 (17.9, 26.3)	19.8 (16.6, 23.1)	18.9 (17.4, 20.4)
Tubers and starchy vegetables	77.7 (72.4, 82.9)	75.1 (72.2, 78.1)	75.0 (69.9, 80.0)	71.1 (65.7, 76.6)	71.5 (66.9, 76.0)	70.3 (67.6, 73.0)	65.9 (61.8, 70.1)	63.7 (60.7, 66.8)	68.1 (63.3, 72.9)	67.7 (63.3, 72.2)
Dairy	268.7 (257.1, 280.3)	269.4 (255.3, 283.4)	267.6 (252.5, 282.8)	276.1 (264.7, 287.5)	263.0 (246.6, 279.4)	283.8 (274.8, 292.8)	270.5 (258.6, 282.5)	273.0 (263.9, 282.2)	254.7 (239.5, 269.9)	239.5 (229.5, 249.5)
Unsaturated oils	18.5 (17.4, 19.6)	21.2 (20.3, 22.2)	22.4 (21.5, 23.2)	24.9 (24.0, 25.9)	24.4 (23.4, 25.5)	25.6 (24.8, 26.4)	28.5 (27.7, 29.4)	28.7 (27.9, 29.6)	30.6 (29.7, 31.5)	32.2 (31.4, 33.0)
Moderation										
Eggs	25.9 (24.3, 27.5)	26.5 (25.1, 27.9)	27.6 (25.5, 29.7)	29.0 (27.6, 30.3)	29.9 (28.3, 31.5)	29.4 (27.4, 31.3)	29.9 (28.7, 31.1)	31.7 (29.9, 33.6)	32.9 (30.8, 35.0)	34.9 (32.9, 37.0)
Red and processed meat	112.5 (106.4, 118.6)	111.4 (107.5, 115.3)	112.9 (108.1, 117.7)	112.1 (108.8, 115.4)	107.9 (103.5, 112.4)	108.3 (103.4, 113.3)	106.1 (100.5, 111.7)	103.5 (100.7, 106.3)	103.2 (98.9, 107.5)	100.9 (97.8, 104.0)
Poultry	43.6 (40.2, 47.1)	43.8 (41.1, 46.4)	47.1 (43.3, 51.0)	49.0 (45.5, 52.4)	50.2 (46.8, 53.7)	51.6 (48.1, 55.1)	48.0 (44.3, 51.7)	51.6 (48.6, 54.6)	51.9 (47.9, 55.9)	53.1 (50.4, 55.8)
Saturated fats	55.1 (53.6, 56.6)	52.4 (50.8, 54.1)	53.0 (51.7, 54.4)	46.5 (45.2, 47.8)	45.3 (43.6, 47.0)	43.5 (41.9, 45.1)	41.8 (40.5, 43.1)	41.3 (40.3, 42.2)	40.7 (39.4, 42.1)	41.9 (40.6, 43.1)
Added sugars	108.7 (101.5, 115.8)	103.0 (98.1, 107.9)	92.4 (88.4, 96.3)	87.5 (83.5, 91.4)	86.8 (81.6, 92.1)	84.5 (81.1, 87.9)	83.4 (79.4, 87.4)	81.8 (78.7, 84.8)	77.4 (74.1, 80.7)	78.1 (74.3, 81.9)

### 3.3 Association of PHDI‐US With All‐Cause and Attributable Mortality Risks

Throughout the follow‐up, 7712 deaths were documented. Multiple models, treating PHDI‐US scores as continuous and categorical variables, were developed. The unadjusted model revealed no significant correlation (HR = 1.000, 95% CI: 0.997–1.002, *p* = 0.705). However, adjustments for age, gender, race/ethnicity, education, PIR and marital (Model 1) demonstrated a significant association (HR = 0.993, 95% CI: 0.990–0.995, *p* < 0.001). Subsequent adjustments (Models 2 and 3) for calorie intake, smoking and alcohol consumption, physical activity, BMI and underlying diseases yielded similar results (HR = 0.995 and HR = 0.994, respectively, both *p* < 0.001). Quartile‐based analyses suggested lower all‐cause mortality in higher PHDI‐US categories after covariate adjustment, with evidence of an overall trend across quartiles in adjusted models (Table 4).

**TABLE 4:** Association between Planetary Health Diet Index for the United States (PHDI‐US) score and all‐cause mortality in NHANES.

	Death (*n*)	Mortality model, HR (95% CI), *p*			
		Univariable model^a^ (*n* = 52,385)	Model 1^b^ (*n* = 44,107)	Model 2^c^ (*n* = 35,251)	Model 3^d^ (*n* = 34,845)
Continuous variable	7712	1.000 (0.997, 1.002), 0.705	0.993 (0.990, 0.995), < 0.001	0.995 (0.992,0.998),< 0.001	0.994 (0.991, 0.997), < 0.001
IQR					
Q1	2071	Reference	Reference	Reference	Reference
Q2	1935	0.976 (0.885, 1.075), 0.620	0.940 (0.853, 1.036), 0.212	0.952 (0.839,1.079),0.442	0.949 (0.835, 1.078), 0.418
Q3	1905	0.957 (0.873, 1.051), 0.359	0.832 (0.762, 0.908), < 0.001	0.864 (0.765, 0.976), 0.019	0.866 (0.768, 0.977), 0.019
Q4	1801	0.991 (0.901, 1.091), 0.861	0.759 (0.687, 0.837), < 0.001	0.788 (0.701, 0.886), < 0.001	0.783 (0.695, 0.882), < 0.001
*p* for trend		0.817	< 0.001	< 0.001	< 0.001

Abbreviations: NHANES, National Health and Nutrition Examination Survey; PHDI‐US, Planetary Health Diet Index for the United States.

^a^ Univariable model was not adjusted.

^b^ Model 1 was adjusted for age, gender, race/ethnicity, education, PIR, and marital.

^c^ Model 2 was additionally adjusted for calorie intake, smoke, alcohol consumption, and physical activity.

^d^ Model 3 was additionally adjusted for BMI and underlying diseases.

In analyses attributable mortality, distinct patterns emerged for cardiovascular and cerebrovascular outcomes. For diseases of the heart (*n* = 1985 deaths), no significant association was observed when treating the PHDI‐US score as a continuous variable (HR = 0.997, 95% CI: 0.990–1.003, *p* = 0.362). However, quartile‐based analysis revealed protective trends, with the highest quartile (Q4) showing a 23% reduction in mortality risk compared to the lowest quartile (HR = 0.773, 95% CI: 0.609–0.981, *p* = 0.034), though the overall trend did not reach statistical significance (*p*‐trend = 0.084).

For cerebrovascular diseases (*n* = 414 deaths), a modest inverse correlation was observed in the continuous model (HR = 0.989, 95% CI: 0.980–0.998, *p* = 0.016). Quartile‐based analysis demonstrated a nonlinear relationship: the third quartile (Q3) exhibited a 58% increased risk (HR = 1.584, 95% CI: 1.020–2.460, *p* = 0.041), while no significant associations were observed in Q2 or Q4. A statistically significant trend was noted across quartiles (*p*‐trend = 0.026).

Other notable findings included reduced mortality risks for accidents (continuous HR = 0.980, *p* = 0.026) and all other causes (Q4 HR = 0.753, *p* = 0.014), with significant trend effects (*p*‐trend = 0.001). No significant associations were identified for malignant neoplasms, chronic respiratory diseases, or diabetes (Table 5).

**TABLE 5:** Association between Planetary Health Diet Score and attributable‐cause mortality in NHANES.

	Death (*n*)	Death model, HR (95% CI), *p*				
		Continuous (*n* = 34,845)	IQR (*n* = 34,845)			*p* for trend
			Q2	Q3	Q4	
Diseases of heart	1985	0.997 (0.990, 1.003) 0.362	0.770 (0.603, 0.984) 0.036	0.814 (0.622, 1.067) 0.136	0.773 (0.609, 0.981) 0.034	0.084
Malignant neoplasms	1721	0.996 (0.988, 1.004) 0.308	0.909 (0.713, 1.158) 0.439	0.904 (0.704, 1.162) 0.431	0.842 (0.653, 1.084) 0.182	0.209
Chronic lower respiratory diseases	422	0.993 (0.981, 1.006) 0.286	1.052 (0.621, 1.781) 0.851	1.187 (0.693, 2.032) 0.532	0.927 (0.523, 1.644) 0.795	0.818
Accidents	248	0.980 (0.962, 0.998) 0.026	0.767 (0.362, 1.624) 0.488	0.392 (0.189, 0.812) 0.012	0.532 (0.264, 1.072) 0.078	0.04
Cerebrovascular diseases	414	0.989 (0.980, 0.998) 0.016	1.509 (0.908, 2.506) 0.112	1.584 (1.020, 2.460) 0.041	0.731 (0.451, 1.185) 0.203	0.026
Alzheimer's disease	269	0.996 (0.980, 1.012) 0.631	1.141 (0.555, 2.345) 0.720	0.832 (0.460, 1.505) 0.543	0.953 (0.536, 1.696) 0.871	0.69
Diabetes mellitus	283	0.997 (0.981, 1.014) 0.735	0.988 (0.540, 1.806) 0.968	0.845 (0.391, 1.826) 0.669	0.945 (0.485, 1.840) 0.868	0.816
Influenza and pneumonia	158	0.994 (0.970, 1.018) 0.605	0.722 (0.280, 1.860) 0.500	0.719 (0.299, 1.727) 0.460	0.723 (0.314, 1.664) 0.446	0.562
Nephritis, nephrotic syndrome and nephrosis	180	0.982 (0.953, 1.011) 0.218	0.626 (0.325, 1.206) 0.162	0.581 (0.245, 1.380) 0.218	0.449 (0.167, 1.208) 0.113	0.144
All other causes	2031	0.993 (0.987, 0.998) 0.011	1.121 (0.864, 1.455) 0.389	0.865 (0.684, 1.095) 0.228	0.753 (0.601, 0.944) 0.014	0.001

**FIGURE 5:**
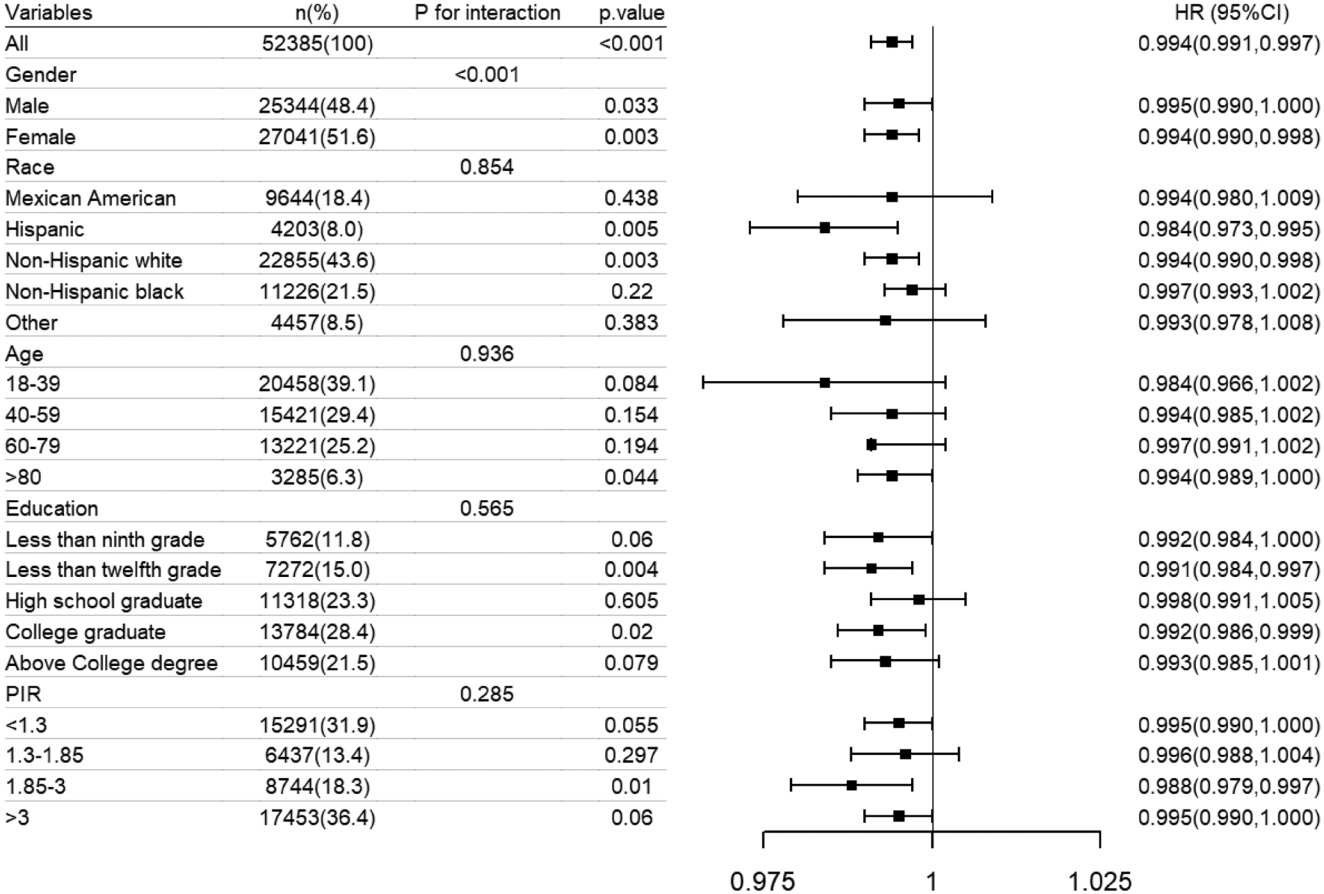
Forest plot of subgroup analysis.

Subgroup analysis, treating PHDI‐US scores as a continuous variable, revealed a pronounced reduction in all‐cause mortality risk associated with increasing PHDI‐US scores. This correlation was especially evident among both male and female, Hispanic and non‐Hispanic white individuals, individuals aged ≥ 80 years, education level from ninth grade to high school graduation or college graduate, those with a poverty‐income ratio (PIR) > 1.85 and < 3. In terms of age, significant interaction effects were observed (Figure 5).

**FIGURE 6:**
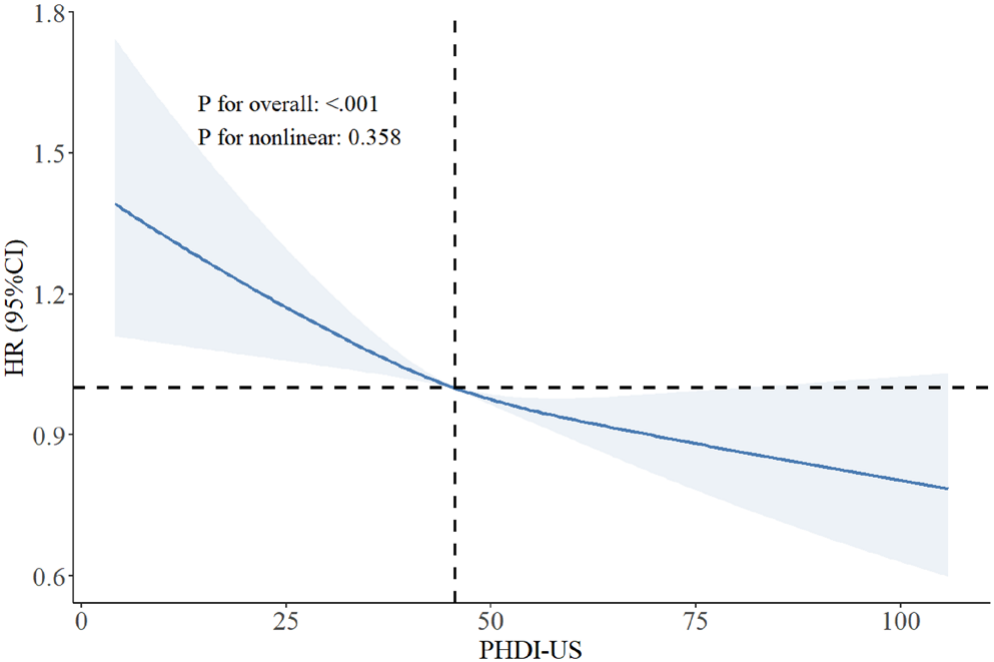
Adjusted RCS curves depicting the relationship between PHDI‐US scores and mortality risk (adjusted for age, gender, race, education, PIR, marital, calorie intake, smoke, alcohol consumption, physical activity, BMI, and underlying diseases).

The relationship between PHDI‐US scores and mortality risk was further delineated using adjusted restricted cubic spline (RCS) curves (Figure 6), adjusted for age, gender, race, education, PIR, marital status, calorie intake, smoking, alcohol consumption, physical activity, BMI, and underlying diseases. The RCS analysis demonstrated a significant overall association (*p* < 0.001) without evidence of significant nonlinearity (*p* = 0.358).

Sensitivity analyses, including multiple imputation, exclusion of the initial survey cycle (1999–2000), and winsorization at the 2nd and 98th percentiles, yielded consistent results, supporting the robustness of the findings (Tables S2–S4 and Figures S1–S3).

## 4 Discussion

This study provides a comprehensive, longitudinal analysis of Planetary Health Diet Index (PHDI‐US) trends and mortality associations among U.S. adults from 1999 to 2020. Overall, PHDI‐US scores increased by 13.8% over 21 years, yet remained low (46.5/150), indicating significant room for improvement toward sustainable dietary targets. Notably, intake of healthful foods (e.g., whole grains) was substantially below, while that of unhealthy components (e.g., red meat) exceeded, EAT‐Lancet recommendations. This study also examined the association between PHDI‐US scores and mortality. After adjusting for age and other covariates, an inverse correlation emerged between the total PHDI‐US score and mortality risk.

PHDI‐US scores showed an overall upward trend from 1999 to 2020 across subpopulations, a pattern consistent with long‐term improvements seen in other dietary quality indexes like the HEI and AHEI (Briefel and Johnson 2004; Wang et al. 2014), and the U.S. Dietary Inflammation Index (DII) (Ryu et al. 2019). The trajectory of PHDI‐US scores offers insight into the sustainability of U.S. diets over time, a finding supported by a separate study using a different index (Frank et al. 2024), with overall scores showing an upward trend from 2003 to 2018. Most of this change occurred before 2011–2012, and scores plateaued thereafter.

Consistent with existing literature on dietary quality, our findings indicate significant differences in scores among racial/ethnic groups. Other races/ethnicities (e.g., Asian) individuals had higher scores, non‐Hispanic white and Hispanic individuals had scores closer to the average, and non‐Hispanic black and Mexican American individuals had the lowest scores. These observations are similar to the findings of He et al. (2021) and other studies (Ma et al. 2022; Tao et al. 2022), underscoring dietary disparities across racial/ethnic groups. Love et al. (2022) have demonstrated that disparities in dietary patterns among racial groups may be influenced by economic affordability. In addition, the results demonstrated that higher average PHDI‐US scores were seen in nonsmokers, women, and older adults, aligning with the findings among Brazilians using the original PHDI (Cacau et al. 2021) and HEI assessments (Guenther et al. 2008, 2014; Reedy et al. 2018).

Certain groups demonstrated greater relative improvements in PHDI‐US scores over the 15‐year period despite not achieving the highest absolute scores by 2017–2020. Significant increases were observed among individuals with less than a ninth‐grade education (8.1%), Mexican Americans (4.1%), males (3.6%), and the highest‐income group (3.5%). This aligns with evidence linking lower socioeconomic status to reduced consumption of healthful plant‐based diets, which emphasize nutritious plant foods like whole grains, fruits, and vegetables (Satija et al. 2017). Lower socioeconomic status may limit consumption of healthier foods (Gonzalgo et al. 2024). The differences in dietary habits among various socioeconomic groups could stem from food insecurity, leading to the consumption of lower‐cost foods; time constraints where parents, due to work, have inadequate time to cook, resulting in children consuming fast food (Zenk et al. 2006).

Our findings align with and extend recent evidence on the PHDI and mortality. For all‐cause mortality, the highest PHDI quartile in our study showed a 22% risk reduction (HR = 0.78, 95% CI: 0.70–0.88), consistent with both Zhan et al. (2025) (HR = 0.65 for Q5 in NHANES) and Bui et al. (2024) (HR = 0.77 for Q5 in pooled cohorts). Similar results were found using another index in a Chinese cohort (HR = 0.85; 95% CI: 0.81–0.89 for the highest quintile) (Ye et al. 2023), despite differences in dietary patterns (e.g., higher plant‐based intake in Asian diets) and scoring methodology (quintiles vs. quartiles). The slightly attenuated effect size compared to Zhan et al. (2025) may reflect methodological differences: their quintile‐based analysis (Q5 vs. Q1) with longer follow‐up versus our quartile‐based approach (Q4 vs. Q1) in a population including individuals with pre‐existing chronic conditions. Despite these variations, all studies adjusted for sociodemographic and lifestyle factors, reinforcing the robustness of the inverse association across diverse populations and dietary indices.

When examining cause‐specific mortality, our results revealed nuanced patterns. For cardiovascular disease, the protective trend in quartile analysis (Q4 HR = 0.773, *p* = 0.034) aligned directionally with Bui et al.'s (2024) stronger quintile‐level association (HR = 0.86 for Q5), though our continuous model lacked significance (HR = 0.997, *p* = 0.362). This discrepancy may stem from differences in population health status (our inclusion of chronic disease patients vs. their exclusion) or statistical power (1985 vs. > 50,000 deaths in Bui et al. (2024)). Notably, both studies observed nonlinear trends (our *p*‐trend = 0.084; their *p*‐trend < 0.0001), suggesting thresholds of dietary adherence may mediate cardiovascular benefits. Furthermore, a previous study examining dietary quality among patients with cardiovascular disease reported an inverse association between PHDI‐US scores and all‐cause mortality (HR = 0.89, *p* = 0.005), findings that are consistent with our results (Pan et al. 2025).

The association between PHDI‐US scores and cerebrovascular mortality presented discrepant patterns across different analytical models. While the continuous model indicated modest protection (HR = 0.989, *p* = 0.016), quartile analysis revealed an unexpected 58% risk increase in Q3 (HR = 1.584, *p* = 0.041)—a finding absent in prior PHDI literature. This contrasts with Bui et al.'s (2024) linear reduction in neurodegenerative mortality (HR = 0.72 for Q5), potentially reflecting outcome‐specific mechanisms or confounding by unmeasured factors like regional sodium intake patterns. The relatively small number of cerebrovascular deaths (*n* = 414) may have contributed to instability in the risk estimates for specific quartiles. Therefore, while a modest overall trend was observed, the inconsistent results across quartiles necessitate a cautious interpretation, and this association requires confirmation in larger studies.

Novel insights emerged for nontraditional outcomes: higher PHDI‐US adherence was associated with reduced mortality from accidents (HR = 0.980, *p* = 0.026) and “other causes” (Q4 HR = 0.753, *p* = 0.014). However, these associations should be interpreted with extreme caution. Unlike outcomes with established biological links to diet (e.g., cardiovascular disease), the observed associations with accidental deaths are far more likely to be explained by residual confounding or unmeasured lifestyle factors (e.g., general risk aversion, socioeconomic status, or other health‐seeking behaviors that are correlated with both healthier diets and lower accident risk), rather than a direct biological effect of diet itself. Therefore, these findings are hypothesis‐generating and require rigorous confirmation in studies specifically designed to control for these potential confounders. Notably, the risk estimates for “other causes” of mortality fluctuated across quartiles and were not consistently significant. The observed statistically significant *p* for trend, in the context of these heterogeneous quartile‐specific estimates, should be interpreted descriptively. It indicates a general pattern of decreasing risk with increasing PHDI‐US scores at the population level, but does not imply a uniform, linear dose–response relationship across all individuals or sub‐groups. This pattern may help identify potential population‐level associations for further investigation.

Zhan et al. (2025) and Bui et al. (2024) emphasized environmental co‐benefits, with Zhan et al. (2025) quantifying a 25% GHG emission reduction in high PHDI adherents. While our study did not assess environmental metrics, the observed mortality reductions strengthen the dual rationale for planetary health diets. Collectively, these findings underscore the need for standardized PHDI scoring and environmental impact assessments to facilitate cross‐study synthesis.

In addition, Zhang et al. (2023) and Gicevic et al. (2021) observed that higher dietary quality scores were associated with lower risks of all‐cause mortality. Similarly, Ye et al. (2023) and Springmann et al. (2018) reported inverse correlations between dietary scores that assessed adherence to the Planetary Health Diet and another proposed sustainable dietary pattern, respectively, and mortality, both overall and from specific causes. Studies like the Malmo Diet and Cancer Cohort and the EPIC study have also documented significant mortality reductions in populations adhering to healthier diets (Stubbendorff et al. 2022; Laine et al. 2021).

This study is not without limitations. First, dietary recalls are subject to potential reporting bias, though NHANES employs the automated multiple‐pass method to mitigate this issue (Briefel and Johnson 2004). Second, the cross‐sectional design of each NHANES survey cycle precludes longitudinal assessment of within‐individual changes in dietary quality over time, does not permit causal inference and can only identify associations between variables, and may be subject to residual confounding due to unmeasured factors. Third, while pooling multiple survey cycles enhanced statistical power, aggregating data across years (Table 1) may obscure temporal variations in dietary patterns. For instance, secular trends like increased processed meat consumption over time‐or shifts in the proportion of processed versus unprocessed meats within composite components (Figure 2) could influence risk estimates but were not explicitly modeled. Fourth, the reliance on all‐cause mortality as the primary outcome has inherent limitations. While morbidity data could theoretically provide a more nuanced understanding of diet‐disease relationships, NHANES data lack sufficient temporal resolution to robustly establish causality for chronic conditions—baseline prevalent diseases may reflect reverse causality (e.g., illness‐driven dietary changes) rather than diet‐induced health effects. Fourth, the estimates for certain cause‐specific mortality outcomes may be statistically unstable due to the limited number of cases. Furthermore, our study sample included participants with two complete dietary recalls as well as those with only one recall. This heterogeneity in dietary assessment may have introduced measurement error in the estimation of PHDI‐US scores, which could have influenced the study findings. Lastly, although this study assessed adherence to the Planetary Health Diet, its primary findings focus on health outcomes. The link between diet and sustainability is inferred indirectly based on the conceptual framework of the index rather than through direct measurement of environmental impact indicators.

To address other potential biases, we adjusted for key confounders including age, gender, race, socioeconomic status (education, PIR), and health‐related factors (BMI, alcohol, and smoking). Strengths include the use of the validated PHDI‐US index (Parker et al. 2023), analysis of 21‐year longitudinal trends in diet quality, mortality risk assessment, and robust sensitivity analyses with consistent findings. Future studies could strengthen causal inference by incorporating two complementary approaches: (1) enhanced temporal modeling of existing NHANES data through stratification by time periods or dynamic weighting of dietary components to better capture secular trends, and (2) prospective cohorts with repeated dietary and health assessments to directly link diet quality to chronic disease trajectories using morbidity data.

Conrad et al. (2023) quantitatively demonstrated that U.S. dietary patterns exhibit distinct sustainability trade‐offs: while plant‐based diets achieved the lowest greenhouse gas emissions (3.5 kg CO_2_eq/day) and food costs ($11.51/day), low‐fat diets showed superior nutritional quality (HEI‐2015 score: 52.0). These evidence‐based comparisons provide policymakers with actionable metrics to balance environmental, economic, and health priorities in national nutrition strategies.

To improve adherence to the Planetary Health Diet (PHD), public policies should address structural barriers in food systems through multisectoral coordination (Swinburn et al. 2019; Springmann et al. 2020). For producers, regulations should prioritize reducing subsidies for environmentally intensive livestock production and incentivize plant‐based agricultural practices. As evidenced by Sweden and Germany's national dietary guidelines, integrating sustainability principles (e.g., reducing red meat consumption and promoting plant‐based foods) requires overcoming lobbying pressures from the beef, dairy, and ultra‐processed food industries (Swinburn et al. 2019). For consumers, Brazil's success in linking dietary guidelines to sustainability goals—which contributed to an 8% decline in urban red meat consumption—demonstrates the potential of policy‐driven behavior change (Swinburn et al. 2019). Furthermore, revising national guidelines to limit animal‐source foods (particularly beef and dairy) while increasing whole grains, legumes, nuts, and vegetables could yield dual health and environmental benefits, though current evidence relies on observational data with moderate certainty.

## 5 Conclusions

The PHDI‐US scores within the U.S. population demonstrate that average diets are far from meeting the healthy and sustainable dietary recommendations outlined in the Planetary Health Diet, with only a small upward trajectory observed over 1999–2020. This trend, consistent across various subpopulations, is accompanied by a decrease in mortality risk associated with higher PHDI‐US scores. Innovative interventions are needed to shift U.S. diets toward aligning with the EAT‐Lancet recommendations, in order to improve food system sustainability and human health.

## Author Contributions

**Mengshan Pan:** conceptualization, methodology, validation, investigation, writing – original draft, writing – review and editing. **Feiyun Zhu:** supervision, writing – original draft, writing – review and editing. **Rucheng Chen:** writing – review and editing, writing – original draft. **Valisa E. Hedrick:** writing – original draft, writing – review and editing, supervision. **Ying Yang:** writing – original draft, writing – review and editing. **Tongle Yin:** conceptualization, methodology, software, data curation, validation, formal analysis, visualization, writing – original draft, writing – review and editing. **Molly K. Parker:** conceptualization, software, supervision, resources, visualization, writing – original draft, writing – review and editing. **Jiamin Xu:** conceptualization, supervision, writing – original draft, writing – review and editing. **ZiYing Jiang:** conceptualization, software, data curation, formal analysis, writing – original draft, writing – review and editing. **Weijun Zheng:** writing – original draft, writing – review and editing, supervision, resources.

## Ethics Statement

Ethical approval and review were not sought or obtained for this study given that it was secondary analysis of publicly available, de‐identified data.

## Conflicts of Interest

The authors declare no conflicts of interest.

## Supporting information


**Figure S1:** RCS curves depicting the relationship between PHDI‐US scores and mortality risk.
**Figure S2:** Adjusted RCS curves depicting the relationship between PHDI‐US scores and mortality risk (adjusted for total energy intake).
**Figure S3:** Adjusted RCS curves depicting the relationship between PHDI‐US scores and mortality risk (adjusted for age, gender, race, EDU, PIR, smoke, drink, and BMI).
**Table S1:** Trends in PHDI‐US scores across years (adjusted for total energy intake).
**Table S2:** Association between Planetary Health Diet Score and all‐cause mortality in NHANES (multiple imputation).
**Table S3:** Association between Planetary Health Diet Score and all‐cause mortality in NHANES (excluding data with follow‐up < 12 months).
**Table S4:** Association between Planetary Health Diet Index for the United States score (winsorized at the 2nd and 98th percentiles) and all‐cause mortality in NHANES.

## Data Availability

The datasets analyzed during the current study are publicly available from the National Health and Nutrition Examination Survey (NHANES) database at https://www.cdc.gov/nchs/nhanes/.
