# Supplementary material for: Trends in Planetary Health Diet Index for the United States (PHDI‐US) Scores and Associations With Mortality Risk in the United States Between 1999 and 2020
Source: Food Sci Nutr. 2026 Jun 11;14(6):e71985. doi: 10.1002/fsn3.71985 (PMC13259960; doi:10.1002/fsn3.71985)
Supplement: Supplementary file 1 — Figure S1: RCS curves depicting the relationship between PHDI‐US scores and mortality risk. Figure S2: Adjusted RCS curves depicting the relationship between PHDI‐US scores and mortality risk (adjusted for total energy intake). Figure S3: Adjusted RCS curves depicting the relationship between PHDI‐US scores and mortality risk (adjusted for age, gender, race, EDU, PIR, smoke, drink, and BMI). Table S1: Trends in PHDI‐US scores across years (adjusted for total energy intake). Table S2: Association between Planetary Health Diet Score and all‐cause mortality in NHANES (multiple imputation). Table S3: Association between Planetary Health Diet Score and all‐cause mortality in NHANES (excluding data with follow‐up < 12 months). Table S4: Association between Planetary Health Diet Index for the United States score (winsorized at the 2nd and 98th percentiles) and all‐cause mortality in NHANES. [file FSN3-14-e71985-s001.docx]

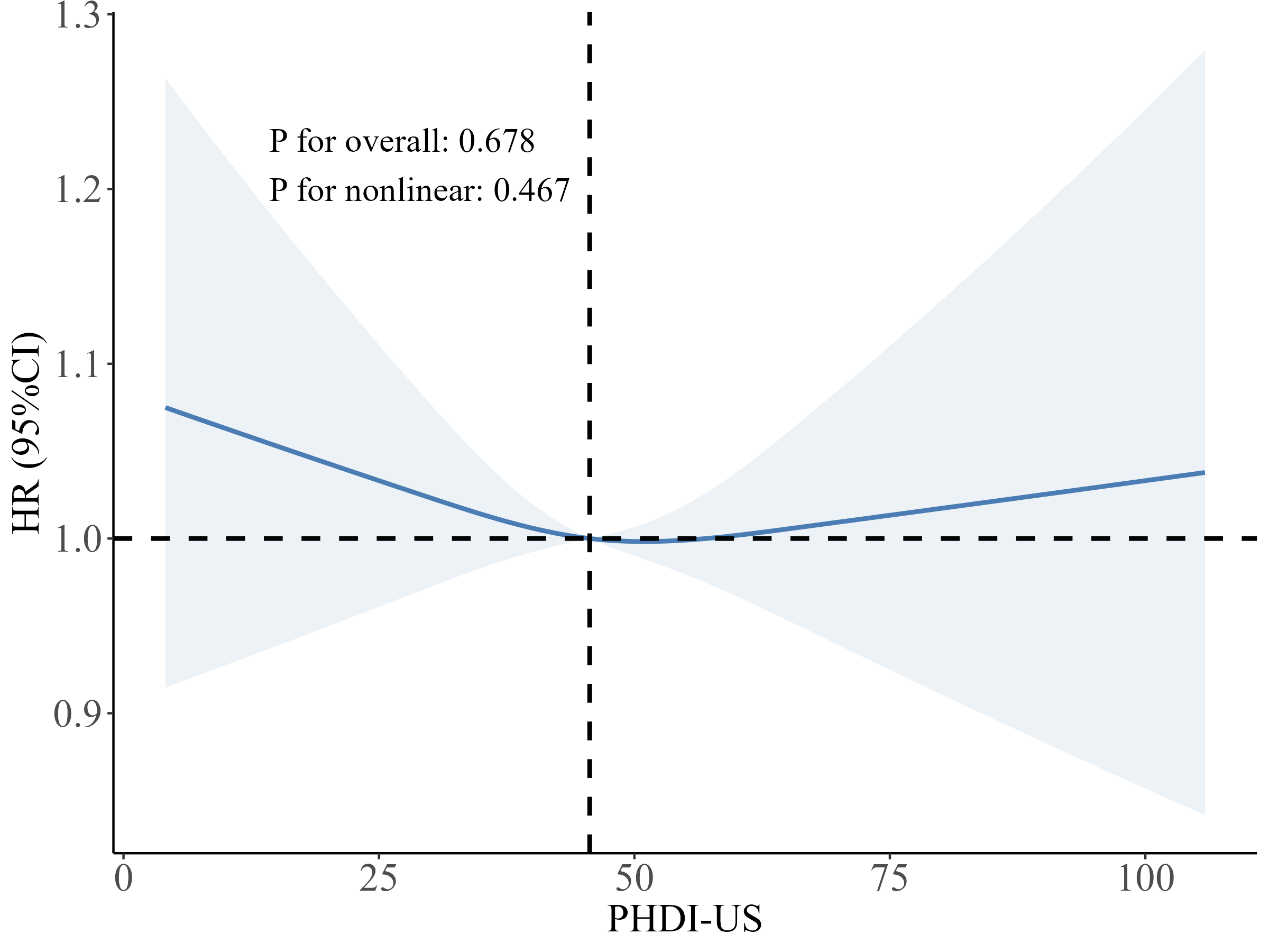


**Supplemental Figure 1.** RCS Curves Depicting the Relationship Between PHDI-US Scores and Mortality Risk.


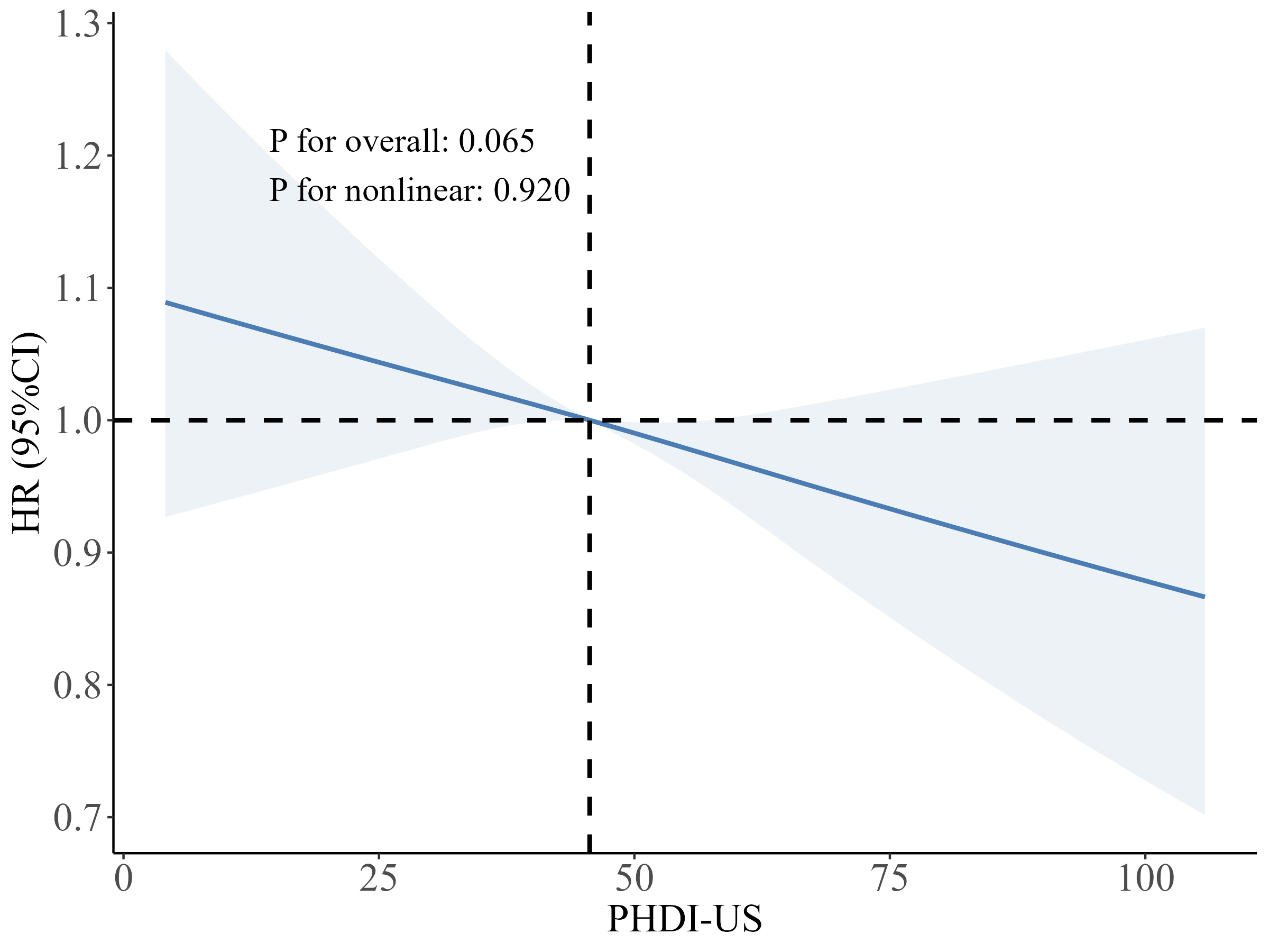


**Supplemental Figure 2.** Adjusted RCS Curves Depicting the Relationship Between PHDI-US Scores and Mortality Risk. (Adjusted for Total energy intake).


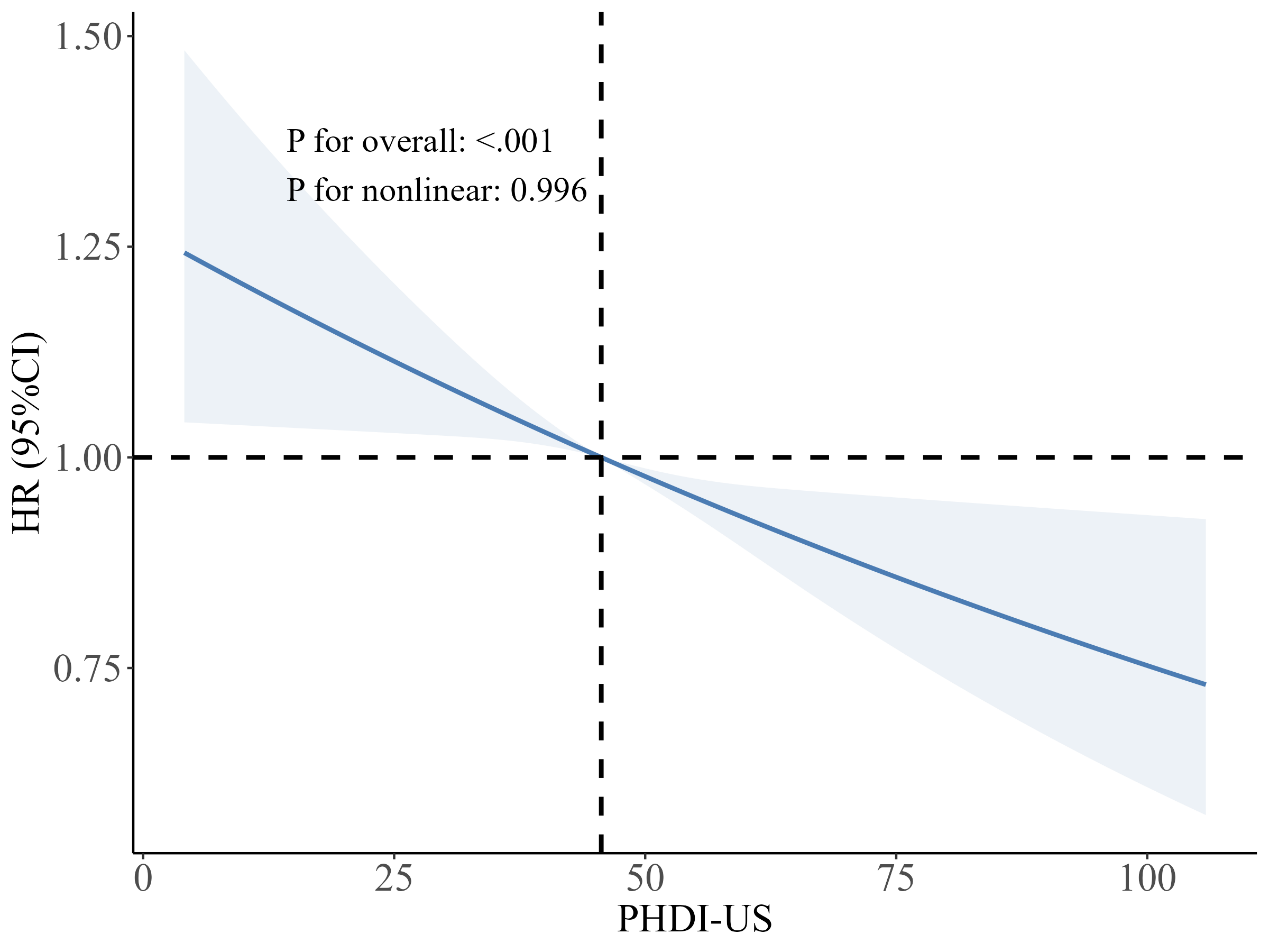
 **Supplemental Figure 3.** Adjusted RCS Curves Depicting the Relationship Between PHDI-US Scores and Mortality Risk. (Adjusted for age, gender, race, EDU, PIR, Smoke, Drink, BMI).

**Supplemental Table 1.** Trends in PHDI-US scores across years(adjusted for Total energy intake).

|  | 1999-2000 | 2001-2002 | 2003-2004 | 2005-2006 | 2007-2008 | 2009-2010 | 2011-2012 | 2013-2014 | 2015-2016 | 2017-2020 | Differences 2017–2020 vs. 1999–2000 (95% CI) | *P* For Trend |
| --- | --- | --- | --- | --- | --- | --- | --- | --- | --- | --- | --- | --- |
| General population | 41.3 (40.0,42.6) | 42.9 (41.9,43.8) | 44.4 (43.6,45.2) | 46.4 (45.4,47.4) | 45.8 (44.6,47.0) | 47.1 (46.5,47.8) | 47.9 (46.8,48.9) | 47.5 (46.6,48.3) | 47.4 (46.1,48.7) | 46.6 (45.7,47.6) | 5.7 (4.1,7.3) | <0.001 |

**Supplemental Table 2.** Association Between Planetary Health Diet Score and All-Cause Mortality in NHANES (Multiple Imputation) .

|  | Death(n) | Mortality model，HR (95%CI), *P* | | | |
| --- | --- | --- | --- | --- | --- |
|  |  | Univariable Model^1^  (n=52385) | Model1^2^  (n=52385) | Model2^3^  (n=52385) | Model3^4^  (n=52385) |
| Continuous variable | 7712 | 1.000(0.998,1.001),0.705 | 0.992(0.990,0.994),<0.001 | 0.995(0.993,0.997),<0.001 | 0.995(0.993,0.997),<0.001 |
| IQR |  |  |  |  |  |
| Q1 | 2071 | Reference | Reference | Reference | Reference |
| Q2 | 1935 | 0.976(0.908,1.049),0.620 | 0.939(0.873,1.010),0.171 | 0.973(0.905,1.046),0.549 | 0.967(0.900,1.040),0.470 |
| Q3 | 1905 | 0.957(0.890,1.030),0.359 | 0.818(0.760,0.881),<0.001 | 0.863(0.801,0.930), <0.001 | 0.861(0.800,0.928),<0.001 |
| Q4 | 1801 | 0.991(0.921,1.067),0.861 | 0.765(0.708,0.826),<0.001 | 0.850(0.786,0.918), <0.001 | 0.847(0.784,0.916), <0.001 |
| *P* for trend |  | 0.817 | <0.001 | <0.001 | <0.001 |

^1^Univariable model was not adjusted.

^2^Model 1 was adjusted for Age, Gender, Race/ethnicity, Education, PIR and Marital.

^3^Model 2 was additionally adjusted for Calorie Intake, Smoke, Alcohol Consumption and Physical Activity.

^4^Model 3 was additionally adjusted for BMI and Underlying Diseases.

**Supplemental Table 3.** Association Between Planetary Health Diet Score and All-Cause Mortality in NHANES (Excluding Data with follow-up <12 months).

|  | Death(n) | Mortality model，HR (95%CI), *P* | | | |
| --- | --- | --- | --- | --- | --- |
|  |  | Univariable Model^1^  (n=51913) | Model1^2^  (n=43685) | Model2^3^  (n=34964) | Model3^4^  (n=34582) |
| Continuous variable | 7243 | 1.000 (0.998,1.003), 0.724 | 0.993 (0.990,0.996), <0.001 | 0.995 (0.992,0.998), 0.003 | 0.995 (0.992,0.998), 0.002 |
| IQR |  |  |  |  |  |
| Q1 | 1930 | 1.00 (reference) | 1.00 (reference) | 1.00 (reference) | 1.00 (reference) |
| Q2 | 1800 | 0.978 (0.884,1.082), 0.663 | 0.940 (0.848,1.042), 0.238 | 0.960 (0.843,1.093), 0.537 | 0.958 (0.839,1.092), 0.519 |
| Q3 | 1803 | 0.977 (0.890,1.073), 0.633 | 0.846 (0.773,0.926), <0.001 | 0.870 (0.769,0.983), 0.025 | 0.871 (0.771,0.984), 0.027 |
| Q4 | 1710 | 1.022 (0.924,1.132), 0.668 | 0.775 (0.698,0.860), <0.001 | 0.806 (0.714,0.910), <0.001 | 0.805 (0.711,0.912), <0.001 |
| *P* for trend |  | 0.65 | <0.001 | <0.001 | <0.001 |

^1^Univariable model was not adjusted.

^2^Model 1 was adjusted for Age, Gender, Race/ethnicity, Education, PIR and Marital.

^3^Model 2 was additionally adjusted for Calorie Intake, Smoke, Alcohol Consumption and Physical Activity.

^4^Model 3 was additionally adjusted for BMI and Underlying Diseases.

**Supplemental Table 4.** Association Between Planetary Health Diet Index for the United States Score (Winsorized at the 2nd and 98th Percentiles) and All-Cause Mortality in NHANES.

|  | Death (n) | Mortality model，HR (95% CI), *P* | | | |
| --- | --- | --- | --- | --- | --- |
|  |  | Univariable Model^1^  (n=52385) | Model 1^2^  (n=44107) | Model 2^3^  (n=35251) | Model 3^4^  (n=34845) |
| Continuous variable | 7712 | 1.000 (0.997,1.002), 0.790 | 0.992 (0.989,0.995), <0.001 | 0.994 (0.991,0.997), <0.001 | 0.994 (0.991,0.997), <0.001 |
| IQR |  |  |  |  |  |
| Q1 | 2071 | 1.00 (reference) | 1.00 (reference) | 1.00 (reference) | 1.00 (reference) |
| Q2 | 1935 | 0.976 (0.885,1.075), 0.620 | 0.940 (0.853,1.036), 0.212 | 0.952 (0.839,1.079), 0.442 | 0.949 (0.835,1.078), 0.418 |
| Q3 | 1905 | 0.957 (0.873,1.051), 0.359 | 0.832 (0.762,0.908), <0.001 | 0.864 (0.765,0.976), 0.019 | 0.866 (0.768,0.977), 0.019 |
| Q4 | 1801 | 0.991 (0.901,1.091), 0.861 | 0.759 (0.687,0.837), <0.001 | 0.788 (0.701,0.886), <0.001 | 0.783 (0.695,0.882), <0.001 |
| *P* for trend |  | 0.806 | <.001 | <.001 | <.001 |

Abbreviations: NHANES: National Health and Nutrition Examination Survey; PHDI-US：Planetary Health Diet Index for the United States

^1^Univariable model was not adjusted.

^2^Model 1 was adjusted for Age, Gender, Race/ethnicity, Education, PIR and Marital.

^3^Model 2 was additionally adjusted for Calorie Intake, Smoke, Alcohol Consumption and Physical Activity.

^4^Model 3 was additionally adjusted for BMI and Underlying Diseases.
